# Cellulose/Sodium Polyacrylate Interpenetrating Network Hydrogel with Intrinsic Anti-Freezing Property

**DOI:** 10.3390/polym17070908

**Published:** 2025-03-27

**Authors:** Qianyun Deng, Yang Wang

**Affiliations:** 1 Key Laboratory for Biobased Materials and Energy of Ministry of Education, College of Materials and Energy, South China Agricultural University, 483 Wushan Road, Guangzhou 510642, China; hellodqy@foxmail.com; 2 Guangdong Provincial Key Laboratory of Plant Resources Biorefinery, School of Chemical Engineering and Light Industry, Guangdong University of Technology, Guangzhou 510006, China; 3 Maoming Branch, Guangdong Laboratory for Lingnan Modern Agriculture, Maoming 525000, China

**Keywords:** intrinsic anti-freezing, hydrogel, cellulose, sodium polyacrylate

## Abstract

Generally, small molecule alcohols and concentrated electrolyte ions can be introduced into the medium of hydrogels as anti-freezing agents to achieve significant anti-freezing properties. However, due to the exchange effect with the external environment, the anti-freezing agents may leak or change in composition causing contamination and unstable material performance during use. Here, cellulose and sodium polyacrylate (PAAS) were used to construct interpenetrating network hydrogels, with cellulose comprising up to 63% of the system. Sodium ions and carboxylic acid groups ionized from the polyacrylate network restricting the formation of water clusters through strong hydration and significantly reduced the ice crystal formation temperature. The rigid cellulose networks provided mechanical strength for the hydrogels. The new interpenetrating network hydrogels exhibited a low anti-freezing temperature (lowest at −56.12 °C), a high water content (over 82.5 wt%), and considerable toughness (up to 2.53 MJ m^−3^). The intrinsic anti-freezing hydrogel constructed in this work provides a new reference strategy for expanding the practicability of anti-freezing hydrogels.

## 1. Introduction

Hydrogels are cross-linked hydrophilic polymer networks, which exhibit solid-like mechanical properties and shapes, as well as liquid-like fast diffusion properties [1,2,3,4]. In a matrix of conventional hydrogels, pure water will form large ice crystals or even freeze completely at subzero temperatures. This can lead to a degradation of performance and a loss of functionality of the hydrogel, such as transparency, electrical conductivity, flexibility, migration of medium, and mechanical durability [5,6,7,8]. Therefore, there has been a growing interest in developing anti-freezing hydrogels that can maintain their performance at extremely low temperatures. The conventional strategies to achieve low-temperature anti-freezing hydrogels, includes the introduction of small molecule organics (such as alcohols), ionic salts, or a combination of both [9,10,11,12,13,14,15]. A few studies have achieved ultra-low temperature anti-freeze properties (−45 °C or lower) by adjusting the composition of anti-freezing agents [16]; however, these strategies have disadvantages. Unless the anti-freezing hydrogels are properly packaged, the liquid medium in the material will exchange with the outside environment, resulting in leakage of the anti-freezing agents, or the infiltration/escape of other media components, which can cause a change in the hydrogel’s performance [17,18,19,20]. Moreover, small organic molecules are usually insulated and not conducive to ion migration. Hydrogels with ionic salts have poor conductivity when used as anti-freezing agents. This issue needs to be compensated by significantly increasing the concentration of electrolyte salts [21]. However, excessive salt concentration can lead to precipitation, causing instability of the overall material.

Based on the above considerations, the development of intrinsic anti-freezing hydrogels (IAHs) that rely solely on the polymer skeleton structure, without the addition of anti-freezing agents, has become a feasible solution. Some recent studies have prepared IAHs based on polyacrylic acid or polyacrylamide. The strong association of hydrophilic groups in the polymer network with water molecules, hinders the formation of ice crystals at low temperatures [22,23]. However, their mechanical properties at low temperatures (no lower than −45 °C) are significantly different from those at room temperature (i.e., they become harder and more brittle). The reason being that although the material is not completely frozen at this low temperature, a large amount of ice crystals can begin to form. Furthermore, these IAHs have a low water content (no higher than 60 wt%) to ensure that no water clusters can form by the additional free water molecules.

In this work, the cellulose/PAAS interpenetrating network hydrogels (CPAs) were fabricated through a stepwise construction method. Pure cellulose hydrogel was constructed through chemical crosslinking based on a cellulose solution. The pure cellulose hydrogels were immersed in a sodium acrylic acid solution, and the sodium polyacrylate (PAAS) networks were constructed through thermally induced polymerization. The transparency, mechanical properties, water loss behavior, and conductivity of the CPAs were examined. Specifically, the changes in storage modulus with temperature were tested to determine the temperature at which ice crystals appeared in the CPAs, serving as the anti-freezing temperature of the CPAs. The results showed that sodium ions and carboxylic acid groups ionized from the PAAS network, restricted the formation of water clusters through strong hydration, and significantly reduced the ice crystal formation temperature. The rigid cellulose network provided a considerable mechanical strength for the hydrogel [24]. The studied interpenetrating network hydrogels had an excellent ultra-low anti-freezing temperature (lowest at −56.12 °C), a high water content (over 82.5 wt%), and superior mechanical properties (tensile strength of 2.59 MPa, elongation at break of 196%, and toughness of 2.53 MJ m^−3^). These CPAs exhibited good performance in low-temperature environments, showing great potential as functional material substrates for low-temperature applications.

## 2. Materials and Methods

### 2.1. Materials

Cellulose (cotton linter) with a degree of polymerization (*DP*) of 534 (containing *α*-cellulose no less than 95%) was supplied by Hubei Chemical Fiber Co., Ltd. (Xiangfan, China); it was dried at 60 °C for 48 h to remove any moisture before use. Benzyltrimethyl ammonium hydroxide (BzMe_3_NOH) (40 wt% aqueous solution) and sodium acrylic acid (SAA) were purchased from Sigma Aldrich Co., Ltd. (Shanghai, China). Epichlorohydrin (ECH, 99% metals basis) and ammonium persulfate (APS) were provided by Shanghai Aladdin Biochemical Technology Co., Ltd. (Shanghai, China). *N*,*N*′-methylene-bis-acrylamide (MBA) was purchased from Energy Chemical Co., Ltd. (Shanghai, China). All the reagents, except the cellulose, were of an analytical standard and were used without further purification.

### 2.2. Fabrication of Cellulose/Sodium Polyacrylate Hydrogels (CPAs)

The cellulose was dispersed in an aqueous solution of BzMe_3_NOH (1.88 mol L^−1^) at room temperature, and the resulting mixture was stored at −40 °C for 3 h to ensure complete freezing. Next, the solid was thawed at 25 °C to obtain the cellulose solution with cellulose concentrations ranging from 4% to 6%. The ECH was added to the cellulose solution and was stirred for 15 min at room temperature. The molar ratio of the ECH to the cellulose anhydroglucose (AGU) unit was 0.8. The resulting mixture was centrifuged at a speed of 8000 rpm for 5 min at room temperature to remove air bubbles. The obtained transparent and viscous solution was poured into a poly(tetrafluoroethylene) mold (10 mm in width, 50 mm in length, and 3 mm in height) with a rectangular shape. The solution in the mold was covered and placed at 20 °C for 12 h to create the chemical cross-linked pure cellulose hydrogels. The obtained cellulose hydrogels were thoroughly washed using deionized water to remove any residual BzMe_3_NOH and ECH and were soaked in a mixed solution consisting of specific concentrations (ranged from 4 mol L^−1^ to 6 mol L^−1^) of SAA, 1.6% MBA, and 0.1% APS, at 20 °C for 24 h. The soaked cellulose hydrogels were removed from the solution, covered, and kept in an oven at 70 °C for 2 h to complete the polymerization of the SAA. The resultant hydrogels were thoroughly washed with deionized water to remove the residual reagents or oligomers to obtain the CPAs. The CPAs were marked as xC-ySAA, where x was the concentration of the cellulose in the BzMe_3_NOH aqueous solution, and y was the concentration of the SAA in solution.

### 2.3. Characterization

A UV-2550 ultraviolet-visible spectrophotometer (Shimadzu Corporation, Kyoto, Japan) was used to measure the optical transmittance curves of the CPAs, within the range from 300 to 800 nm. The transmittance at 550 nm was used to represent the transparency within the visible light range of the sample; three parallel samples were tested for each group.

Cross-section and surface SEM images of the freeze-dried CPAs were taken with a field emission scanning electron microscope (FE-SEM) (Zeiss, SIGMA, Oberkochen, Germany) operating at an acceleration voltage of 5 kV.

The tensile tests of the CPAs were performed using an electromechanical Universal Testing Machine (MTS Systems Co., Ltd., Beijing, China) with a 500 N load cell. The deformation rate in the tensile tests was 10 mm min^−1^; five parallel samples were tested for each group. The test samples had a rectangular shape with dimensions of 10 mm in width and 50 mm in length.

The dynamic mechanical behaviors of the CPAs were measured using a dynamic mechanical analyzer (Netzsch DMA 242C, Selb, Germany) in a temperature range from 25 °C to −90 °C, in an inert gas medium with a flow rate of 60 mL min^−1^. The cooling rate was 3 °C min^−1^. In the tensile mode, a static force of 0.5 N and a dynamic force of 0.1 N at the constant frequency of 1 Hz and the amplitude of 1%, were applied to the CPAs. The CPAs were also characterized using a differential scanning calorimeter (NETZCH DSC, Selb, Germany). The DSC was operated under a 60 mL min^−1^ nitrogen flow rate. Samples were first equilibrated at 25 °C, then cooled to −70 °C at a rate of 5 °C min^−1^.

The conductivities of the CPAs were measured by a UC3545 Insulation Resistance Tester (UCE Technologies, Ltd., Changzhou, China) with copper foil as the electrodes. Both ends of the CPAs were wrapped with a thin layer of copper foil and fixed using insulating tape to ensure a good contact; five parallel samples were tested for each group.

The water evaporation tests of the CPAs were carried out under a relative humidity (RH) of around 45%, and a temperature around 30 °C. A saturated K_2_CO_3_ solution was placed in a DZF-6020 vacuum drying oven (Kangheng Instrument Co., Ltd., Guangzhou, China) to establish a constant temperature and RH conditions for the water evaporation tests. The real-time weight of the hydrogel sample was recorded at an hourly interval. The water loss ratio was calculated using Equation (1):

water loss ratio = (*W*_0_ − *W*)/*W*_0_ (1)

where *W* refers to the real-time weight of the CPAs and *W*_0_ represents the initial weight of the CPAs. The CPAs were weighed and recorded until the weight was constant. At least three parallel samples were tested.

The mass ratios of the cellulose network to the PAAS network in the CPAs were estimated through the process of weighing. It was assumed that the cellulose was fully involved in the composition of the CPAs. The CPAs were freeze-dried and the resulting mass was determined. The masses of the APS, ECH, and MBA were negligible. The mass ratio of the cellulose network to the PAAS network was calculated using Equation (2):

*Q* = *m*_1_/(*m*_2_ − *m*_1_) (2)

where *Q* refers to the mass ratio of the cellulose to the PAAS in the CPAs, *m*_1_ was the initial weight of the cellulose, and *m*_2_ was the weight of the dried CPAs.

## 3. Results and Discussion

**Figure 1:**
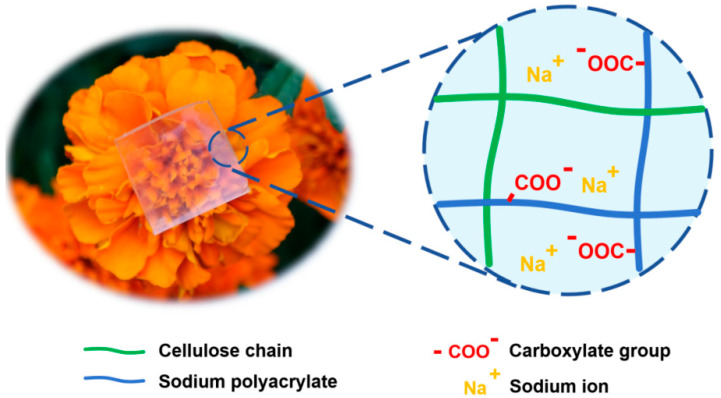
Photograph and polymer network schematic diagram of transparent CPAs (5C-4SAA was taken for example).

The CPAs were prepared in three steps: (1) the cellulose was dissolved in a BzMe_3_NOH solution and the cross-linking agent ECH was added to conduct the chemical cross-linking, which formed the cellulose network; (2) the monomer SAA, the cross-linking agent MBA, and thermal initiator APS were introduced into the pure cellulose hydrogel through simple immersion; and (3) the penetration and cross-linking polymerization of the SAA monomer constructed the network of PAAS. ECH is a commonly used chemical cross-linking agent in alkaline aqueous cellulose solution systems. The cross-linking reaction mechanism is as follows. Under alkaline conditions, the hydroxyl groups on the cellulose chains are deprotonated to form reactive alkoxide ions (-O^−^), which act as nucleophiles. These ions attack the less substituted carbons of the strained epoxide ring in the ECH, opening the ring and forming an intermediate chlorohydrin structure attached to the cellulose. The chlorine atom in this intermediate serves as a leaving group. Subsequently, a second deprotonated hydroxyl group (from another cellulose chain or a different site on the same chain) performs a nucleophilic substitution (SN_2_) on the carbon bonded to the chlorine; this displaces Cl^−^ and forms a stable ether linkage (-O-) between the two AGU units. This process repeats, creating a three-dimensional network of interconnected cellulose chains via ether bonds. Thermal initiated chemical cross-linking polymerization is one of the common methods used to prepare a PAAS hydrogel; the mechanism is as follows. Upon heating, the thermal initiator APS decomposes to generate sulfate radical anions (SO_4_^−•^); these abstract a hydrogen atom from the monomer SAA forming initiating radicals. These radicals attack the vinyl group of the SAA, initiating chain propagation through the sequential addition of the SAA to form growing polymer chains. Simultaneously, the cross-linking agent MBA containing two reactive acrylamide groups participates in the polymerization. The radicals at the ends of the propagating polymer chains react with the vinyl groups on the MBA creating covalent bridges between adjacent polymer chains. As the reaction progresses, chain termination occurs via radical recombination or disproportionation, finalizing the formation of a three-dimensional network. In the schematic diagram of the network structure Figure 1, the CPAs were typical interpenetrating network hydrogels [25,26,27,28,29]. Although there may be hydrogen bonding interactions between the cellulose and the PAAS molecular chains, the processes of forming these two networks were relatively independent. Due to the different molecular chain structures, the rigidity of the cellulose network was relatively strong, while the rigidity of the PAAS network was relatively weak. Forming an interpenetrating network based on such polymer networks has been a typical strategy for creating strong and tough hydrogels. The resulting mechanical properties can be adjusted by the relative proportions of these networks.

**Figure 2:**
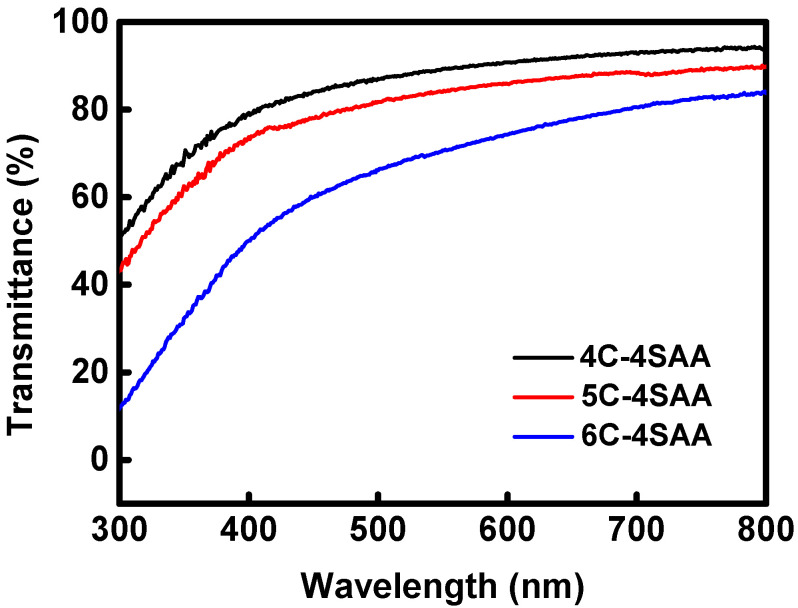
UV-vis transmittance curve of CPAs.

**Figure 3:**
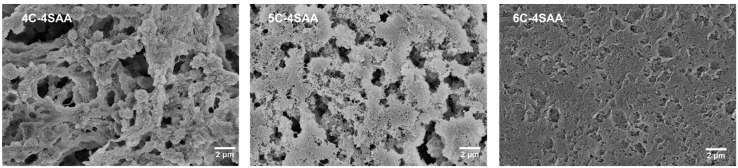
SEM images of cross-sectional morphologies of CPAs.

A photograph of 5C-4SAA is shown in Figure 1, while the relevant ultraviolet-visible transmission curves of the CPAs are displayed in Figure 2 and Table S1. Obviously, for cellulose concentrations no higher than 5%, the CPAs demonstrated a high transparency in the ultraviolet-visible range with a transmittance of over 83.9% at 550 nm. This is because the hydrogel network is composed of cross-linked and well dispersed cellulose and PAAS chains. The high transparency enabled the CPAs to be served as substrates for the optical devices, without hindering the propagation of optical signals while exerting their functionality. However, as the cellulose concentration reached 6%, the polydispersity of the cellulose raw materials led to an increase in the amount of insoluble high molecular chains; this resulted in a decrease in the uniformity of the cellulose network and a decrease in transparency to around 70% at 550 nm. As shown in Figure 3, the CPAs exhibited a porous structure with micrometer-sized pores. The CPAs became denser as the mass ratios of the cellulose network to the PAAS network increased, which was caused by the higher cellulose solution concentrations.

**Figure 4:**
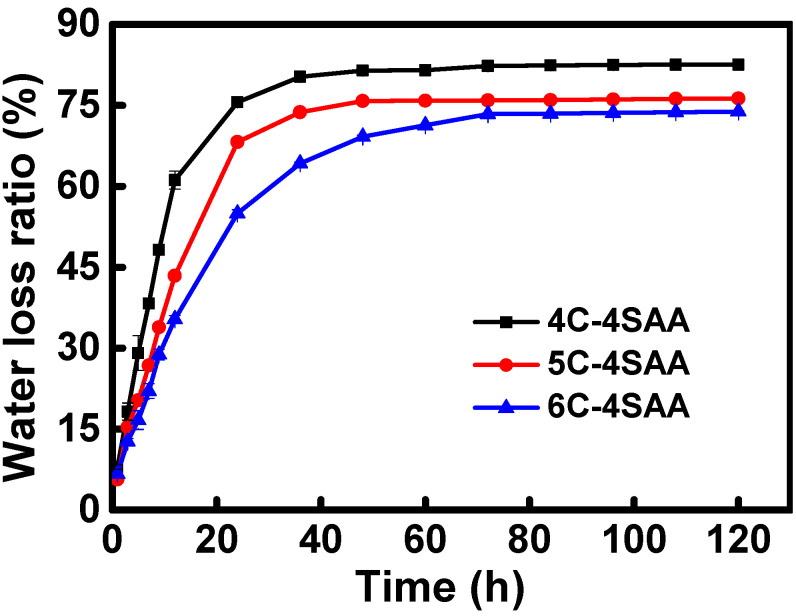
Water loss curves of CPAs at a RH condition of 45% and temperature of 30 °C.

The water loss curves of the CPAs at a RH condition of 45% and a temperature of 30 °C are shown in Figure 4 and Table S2. After 70 h, the CPAs finally reached a state of water loss equilibrium, with the final water loss ratio of the CPAs ranging from 73.8 wt% to 82.5 wt%. This result demonstrates that if the water content had been calculated by completely drying the hydrogel, the maximum water content of CPAs might be above 82.5 wt%, far higher than other reported IAHs. Table 1 is the recipe for the preparation of the CPAs and the corresponding amounts of raw materials. During the process, BzMe_3_NOH, ECH, APS, and water were the only materials used for treatment. Table 2 is the actual composition of the final products. The cellulose content accounts for 40–60% in the final products. With an increase in the cellulose solution concentration, the obtained cellulose hydrogel became more compact and the penetration of the SAA monomer became more difficult, resulting in a decrease in the PAAS network content in the CPAs. In addition, the rigid cellulose network provided a considerable mechanical strength for the hydrogel, which played a very important role for the preparation of the final products. It was observed that as the mass ratios of the cellulose network to the PAAS network increased, the final water loss ratios of the CPAs decreased. This result is consistent with the trend that the CPAs’ structure became denser (shown in Figure 3). Further, this was understandable since the PAAS have a stronger hydrophilicity and better water retention ability compared with cellulose. For the same reason, polyacrylic acid, PAAS, and polyacrylamide are generally used instead of cellulose in existing commercial absorbent resins [30,31,32,33].

**Figure 5:**
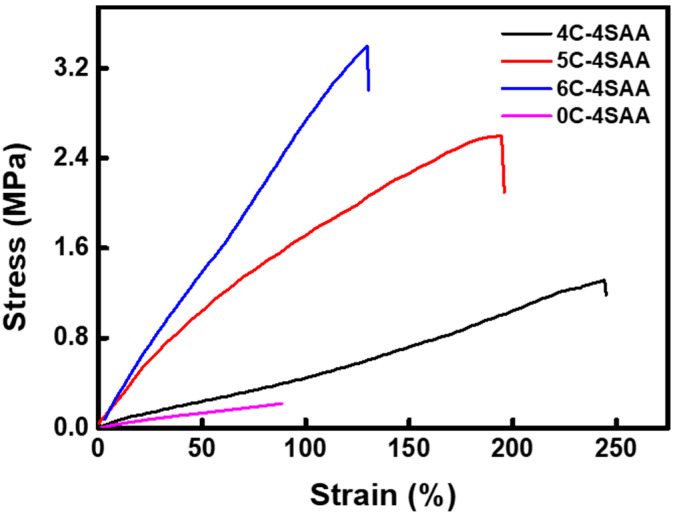
Stress-strain curves of the CPAs, and the pure PAAS hydrogel (0C-4SAA).

In Figure 5 and Table S3, it can be seen that the pure PAAS hydrogel exhibited a poor mechanical property, with a tensile strength of only 0.22 MPa, which can be attributed to the lack of rigidity of the PAAS chain. With an increasing proportion of cellulose, the CPAs demonstrated an increase in tensile strength from 1.31 MPa to 3.40 MPa, and a Young’s modulus from 0.53 MPa to 2.61 MPa, and a gradual decrease in elongation at break from 245% to 130%. All of the CPAs with interpenetrating networks exhibited significantly stronger mechanical properties than the PAAS, due to the introduction of rigid cellulose chains and the hydrogen bonding interactions between the networks. Although cellulose was not the key component according to existing IAHs, the cellulose in this work provided a significant positive promotion to the strength and toughness of the CPAs. It was calculated that 5C-4SAA had the highest toughness of 2.53 MJ m^−3^, which was 28 times higher than that of the pure PAAS hydrogel.

**Figure 6:**
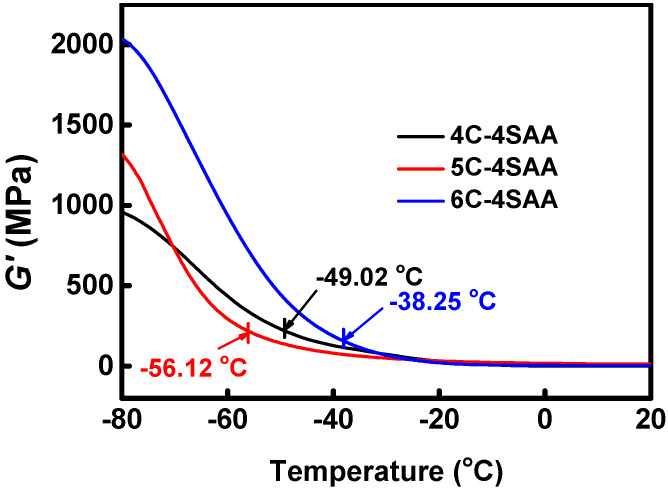
DMA diagram characterization of the CPAs, and the temperatures that ice crystals began to form are marked on the curves of the storage modulus (*G*′).

**Figure 7:**
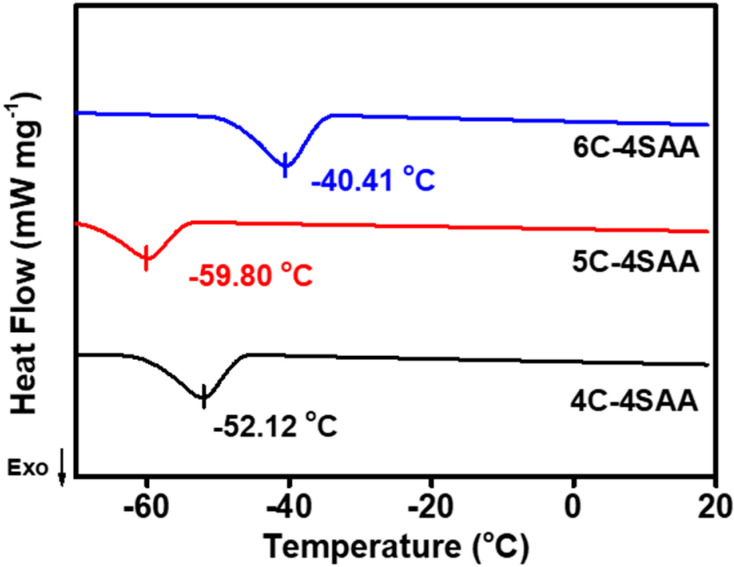
DSC curves of the CPAs from 20 °C to −70 °C.

**Figure 8:**
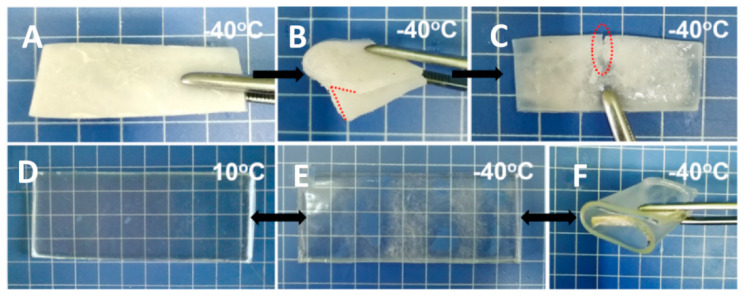
The appearance of the pure cellulose hydrogel, and 5C-4SAA at different temperatures. (**A**–**C**) The pure cellulose hydrogel at −40 °C. (**D**–**F**) 5C-4SAA at 10 °C and −40 °C.

As shown in Figure 1, PAAS is a polyelectrolyte which can be ionized into sodium ions and carboxylate groups, and the cellulose chains have a large number of hydroxyl groups, which can exhibit a strong hydrophilicity. The above ionizable or hydrophilic groups and ions can form strong associations with water, destroy water molecule clusters, and inhibit ice crystal formation. This effect can substantially reduce the freezing point of a medium, which is the same principle as using conventional small molecule anti-freezing agents. The only difference is that in this study, these anti-freezing effects did not come from the free small molecules in the hydrogel medium, but from the polymer network skeleton. It can be calculated from the curves in Figure 6, that the *G*′ of the CPAs are continuously stable from room temperature and began to rise as the temperature goes down to a certain point, which is commonly considered as point at which tiny ice crystals appear. The anti-freezing temperatures of IAHs claimed in the existing research are no lower than −45 °C, at which point the gel materials retain a certain degree of flexibility, electrical conductivity, or transparency, with no obvious icing. However, the actual temperatures at which the ice crystals appear are even higher, and these gels become significantly stiffer due to the appearance of tiny ice crystals. For different CPAs, the temperatures that ice crystals begin to form ranges from −38.25 °C to −56.12 °C, at which temperatures the CPAs are not completely frozen. In Figure 7, the exothermic peak corresponds to the universal generation of ice crystals appearing on each sample curve. This temperature is slightly lower than the temperature at which the ice crystals begin to form on a small scale in Figure 6. The above results indicate that the CPAs maintained stable and unchanged mechanical properties from room temperature to extreme low temperatures, and the CPAs have an ultra-low anti-freezing temperature with a minimum of nearly −60 °C. Figure 8 provides a more intuitive representation of the anti-freezing property of 5C-4SAA at a low temperature. Water in the pure PAAS hydrogel froze (0C-4SAA) (Figure 8A–C), leading to an even turbid appearance, and a permanent deformation and fracture upon bending. In contrast, 5C-4SAA (Figure 8D,E) maintained elasticity and transparency at both 10 °C and −40 °C and could be reversibly curled without fracture (Figure 8F). In summary, the CPAs in this work (especially 5C-4SAA) exhibited superior anti-freezing properties compared with the existing reported IAHs.

**Figure 9:**
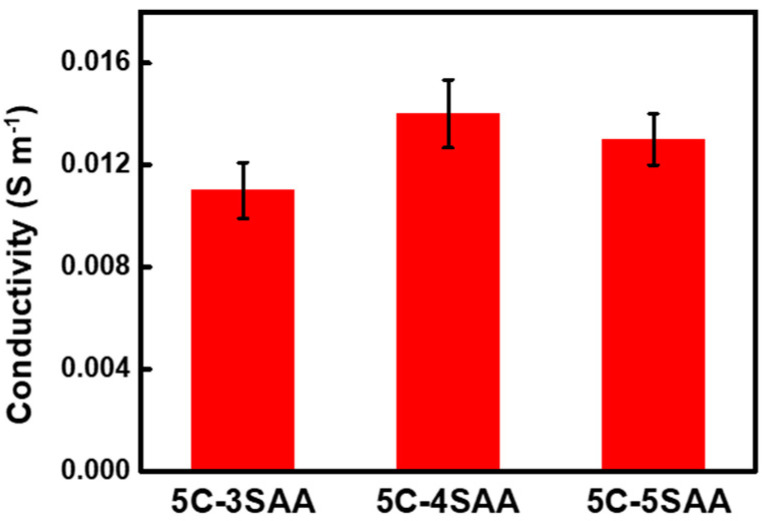
Conductivity of the CPAs.

Based on the above results, the CPAs prepared using a 5% cellulose solution exhibited excellent transparency, mechanical properties, and anti-freezing properties. Generally, hydrogels with electrolyte ions as anti-freezing agents will have both anti-freezing properties, and a degree of conductivity. Here, the electrical conductivities of the CPAs prepared from different concentrations of the SAA monomer solutions were investigated. As shown in Figure 9 and Table S4, the conductivities of the CPAs were found to be quite low, within the range from 0.011 S m^−1^ to 0.014 S m^−1^. The reason is the sodium ions were bound by the charge interaction of the carboxyl groups, and their migration ability was limited, which also meant that the anti-freezing agents were difficult to cause leakage through migration.

## 4. Conclusions

In summary, this work presented a method for the preparation of CPA interpenetrating network hydrogels with ultra-low temperature intrinsic anti-freezing properties, and a high water content. The cellulose network and the PAAS network in the CPAs were independently constructed in a stepwise fashion. Among the CPAs, 5C-4SAA exhibited an impressive anti-freezing temperature (temperatures that ice crystals began to form) of −56.12 °C, superior to existing IAHs. The ionizable (-COO^−^) or hydrophilic groups (-OH), and ions (Na^+^) on the cellulose and PAAS chains, could form strong associations with water, destroying water molecule clusters, and inhibiting ice crystal formation, thereby remarkably reducing the freezing point of the medium. The CPAs in this work had the anti-freezing property at ultra-low temperatures and maintained their mechanical properties consistent with room temperature performance. Furthermore, as a type of IAH, the CPAs avoided leakage, or changes in the composition of the anti-freezing agents. Additionally, 5C-4SAA demonstrated a high water content (>82.5 wt%), high transparency (83.9%), and favorable mechanical properties, including a tensile strength of 2.59 MPa, elongation at break of 196%, and a toughness of 2.53 MJ m^−3^. This work proposes a construction method of an IAH with excellent anti-freezing properties and a comprehensive performance, which provides a feasible idea for the preparation and practical utilization of hydrogels in extremely cold environments. Future studies should focus on enhancing the conductivity of CPAs through the incorporation of conductive nanomaterials, broadening their applicability in flexible electronics and low-temperature sensors. Exploring the long-term stability of CPAs under cyclic freeze-thaw conditions or mechanical stress would further validate their reliability in real-world scenarios, such as polar exploration equipment or cryogenic biomedical devices. Additionally, evaluating the biodegradability and biocompatibility of these hydrogels may unlock their potential in tissue engineering or drug delivery systems for cold environments. Efforts to optimize the synthesis process, by reducing chemical solvent usage or adopting greener crosslinking strategies, could align the fabrication of CPAs with sustainable material development goals. Extending this design principle to other polymer networks may inspire novel multifunctional hydrogels tailored for extreme conditions.

## Figures and Tables

**Table 1:** Sample codes of the CPAs and the corresponding amounts of raw materials used in their preparation.

Sample	Cellulose /g	BzMe_3_NOH (1.88 mol L^−1^ aq.) /g	ECH /g	MBA /g	APS /g	Sodium Acrylate /g	Water /g
4C-4SAA	0.40	10.00	0.18	5.50	0.30	94.00	250.00
5C-4SAA	0.50	10.00	0.23	5.50	0.30	94.00	250.00
6C-4SAA	0.60	10.00	0.27	5.50	0.30	94.00	250.00
5C-3SAA	0.50	10.00	0.23	5.14	0.30	71.00	250.00
5C-5SAA	0.50	10.00	0.23	5.89	0.30	118.00	250.00
0C-4SAA	0	10.00	0.00	5.50	0.30	94.00	250.00

**Table 2:** The mass ratios of the cellulose network to the PAAS network in the CPAs.

Sample	Cellulose Network: PAAS Network
4C-4SAA	4:5.52
5C-4SAA	5:4.64
6C-4SAA	6:3.51

## Data Availability

The original contributions presented in the study are included in the article, further inquiries can be directed to the corresponding author.

## Supplementary Materials

The following supporting information can be downloaded at: https://www.mdpi.com/article/10.3390/polym17070908/s1, Table S1: The transmittance of the CPAs at 550 nm; Table S2: The water loss rate of the CPAs when they reached the equilibrium state of water evaporation; Table S3: The mechanical data of the CPAs; Table S4: Conductivity of the CPAs.
