# Supplementary material for: Cellulose/Sodium Polyacrylate Interpenetrating Network Hydrogel with Intrinsic Anti-Freezing Property
Source: Polymers (Basel). 2025 Mar 27;17(7):908. doi: 10.3390/polym17070908 (PMC11991314; doi:10.3390/polym17070908)
Supplement: Supplementary file 1 [file polymers-17-00908-s001.zip › polymers-3540193-supplementary.pdf]

# Cellulose/Sodium Polyacrylate Interpenetrating Network Hydrogel with Intrinsic Anti-Freezing Property

**Qianyun Deng [1,2] and Yang Wang [1,3,*]**

[1] Key Laboratory for Biobased Materials and Energy of Ministry of Education, College of Materials and Energy, South China Agricultural University, 483 Wushan Road, Guangzhou 510642, China
[2] Guangdong Provincial Key Laboratory of Plant Resources Biorefinery, School of Chemical Engineering and Light Industry, Guangdong University of Technology, Guangzhou 510006, China
[3] Maoming Branch, Guangdong Laboratory for Lingnan Modern Agriculture, Maoming 525000, China

**Table S1.** The transmittance of the CPAs at 550 nm.

**Table S2.** The water loss rate of the CPAs when they reached the equilibrium state of water evaporation.

**Table S3.** The mechanical data of the CPAs.

**Table S4.** Conductivity of the CPAs.

**Table S1.** The transmittance of the CPAs at 550 nm.

| Sample | Transmittance (%) |
|---|---|
| 4C-4SAA | 89.3 |
| 5C-4SAA | 83.9 |
| 6C-4SAA | 70.7 |

**Table S2.** The water loss rate of the CPAs when they reached the equilibrium state of water evaporation.

| Sample | Water loss rate (%) |
|---|---|
| 4C-4SAA | 82.5±0.5 |
| 5C-4SAA | 76.2±0.4 |
| 6C-4SAA | 73.8±0.1 |

**Table S3.** The mechanical data of the CPAs.

| Sample | Tensile strength (MPa) | Elongation at break (%) | Toughness (MJ $m^{-3}$) | Young's modulus (MPa) |
|---|---|---|---|---|
| 4C-4SAA | 1.31 | 245 | 1.60 | 0.53 |
| 5C-4SAA | 2.59 | 196 | 2.53 | 1.32 |
| 6C-4SAA | 3.40 | 130 | 1.91 | 2.61 |
| 0C-4SAA | 0.22 | 88 | 0.09 | 0.26 |

**Table S4.** Conductivity of the CPAs.

| Sample | Conductivity (S $m^{-1}$) |
|---|---|
| 5C-3SAA | 0.011±0.0011 |
| 5C-4SAA | 0.014±0.0013 |
| 5C-5SAA | 0.013±0.0010 |
